# The effects of CuO/CeO_2_ mixture nanoparticles on the performance of a vapor compression refrigeration system

**DOI:** 10.1038/s41598-022-12942-7

**Published:** 2022-05-25

**Authors:** HudaElslam Abdali Mohamed, Unal Camdali, Atilla Biyikoglu, Metin Aktas

**Affiliations:** 1 Department of Mechanical Engineering, Ankara Yildirim Beyazit University, 06010 Ankara, Turkey; 2 Department of Mechanical Engineering, Gazi University, 06010 Ankara, Turkey; 3 Department of Energy, Ankara Yildirim Beyazit University, 06010 Ankara, Turkey

**Keywords:** Energy science and technology, Engineering, Nanoscience and technology

## Abstract

This study was built on the basis of experimental results from a simple refrigeration system using R134a as a refrigerant. Based on the real dimensions of the system and the experimental results, Ansys fluent software was used to simulate the system to prepare the system to introduce the nanoparticles theoretically. Since the nanoparticle preparation process is expensive, this research presents a simple, easy, and inexpensive method for the preparation process based on, distilled water, ammonia, copper nitrate, and cerium nitrate to synthesize seven types of nanoparticles as a single oxide and as a mixture from two different oxides The results of preparing using X-ray diffraction and scanning electron microscopy confirmed that the particles were spherical in shape, with suitable average diameters ranging between 78.95 nm, 79.9 nm, 44.15 nm and 63.3 nm for copper oxide, cerium oxide, the first mixture, and the second mixture respectively. The theoretical study confirmed that both copper oxide, cerium oxide, and the mixture consisting of both improved the performance of the refrigeration system and reduced energy consumption. Moreover using the numerical equations available in the literature to calculate the thermophysical properties proved an improvement in these properties with an increase in the nanoparticle concentration when mixed with R134a.

## Introduction

Most of the current studies focus on improving the performance of refrigeration systems and air conditioners, as they are among the most energy consuming sectors. To improve the thermal properties of the working fluid, very small particles, ranging in size from millimeters to micrometers, are dispersed inside the base fluid, which was made by Maxwell in 1873, but this attempt faced many problems, including stability, clogging, and erosion. In the late twentieth century. Choi presented the working fluid in a new concept, where the nanoparticles are dispersed inside the primary fluid to improve its thermal properties^1,2^. The nanofluid is classified as follows (i) mono-nanofluids which consist of similar nanoparticles, (ii) hybrid nanofluids which consist of dissimilar nanoparticles; and (iii) hybrid nanofluids which consist of composite nanoparticles^1^. To achieve the best heat transfer properties between the fluids and the nanoparticles, the following must be provided (i) dispersibility of nanoparticles (ii) stability of nanoparticles (iii) chemical compatibility of nanoparticles and (iv) thermal stability of nanofluids^3^. Recently, the concept of nanofluids has been developed to include refrigerants as nanorefrigerants and lubricant oils as nanolubricants, where the method of preparation is limited to using a one-step method and a two-step method. In the two-step the nanoparticles are manufactured as a powder, and then put into the base fluid, followed by several types of dispersion methods such as agitation either by ultrasonic or magnetic force, homogenizing, and high shear mixing to disperse nanoparticles inside a mixture. A one-step method is based on condensing vapor nanophase powders into liquid by reducing the pressure and then dissolving them inside the liquid immediately^4,5^.

### Literature review

In this section, the latest studies, and results will be presented that include the addition of nanoparticles to refrigeration systems, as well as the effect of nanoparticles on improving the thermophysical properties of the working fluid.

#### Evaluation of the refrigeration system based on nanolubricants

Vijayakumar et al.^6^ studied the effect of nanolubricants on the performance of refrigerators based on aluminum dioxide mixed with polyolester oil, and 60 g R602a was charged as a refrigerant. The results indicated that the improvements in both the cooling capacity and the COP were 6.09%, and 20.09% respectively, while the reduction in the power consumed was 15.78%. Choi et al.^7^ studied the effect of nanolubricants on the performance of refrigerators based on 0.1 wt% MWCNTs was dispersed into the polyolester oil, and R134a was used as a refrigerant. The results indicated that the power consumption was reduced by 17%. Senthilkumar et al.^8^ studied the effect of nanolubricants on the performance of refrigerators based on Al_2_O_3_ and SiO_2_ hybrid nanoparticles and 60 g R600a was used as a refrigerant. The results showed that the improvements in both the COP and cooling capacity were 30 and 25% respectively, while the power consumed was reduced by 80 W. Senthilkumar et al.^9^ studied the effect of nanolubricants on the performance of a vapor compression refrigeration system based on CuO and SiO_2_, and 40 and 60 g R600a were used as refrigerants. The results showed that both the COP and cooling capacity improved by 35% and 18% respectively, while the reduction in the power consumed was 75 W. Senthilkumar et al.^10^ studied the effect of nanolubricants on the performance of refrigeration system based on 0, 0.2, 0.4 and 0.6 g/L SiO_2_ added to polyolester oil and R410A was charged as the refrigerants. The results showed that 0.4 g/L SiO_2_ achieved the best cooling capacity, reduced the power consumed by 80 W, and enhanced COP by 1.7. Senthilkumar et al.^11^ studied the effect of nanolubricants on the performance of the refrigeration system based on 0.4 g/L and 0.6 g/L ZnO/SiO_2_ hybrid nanoparticles, and R600a was used as a refrigerant. The results showed that 0.6 g/L ZnO/SiO_2_ achieved a high cooling capacity of 180 W, and enhanced the COP by 1.7, while the lower power consumed was 78 W. Senthilkumar et al.^12^ studied the effect of nanolubricants on the performance of the refrigeration system based on 0.2, 0.4 and 0.6 g/L of CuO/Al_2_O_3_ hybrid nanoparticles, and 70 g R600a was charged as a refrigerant. The results indicated that the addition of CuO/Al_2_O_3_ improved both the COP and cooling capacity by 27% and 20% respectively, while the reduction in the power consumed was by 24%. Javadi et al.^13^ studied the effect of the nanolubricants on the performance of the refrigerators based on 0.1 wt% Al_2_O_3_. The results showed that 0.1 wt% Al_2_O_3_ reduced the power consumed by 2.69%. Gill et al.^14^ studied the effect of nanolubricants on the performance of a domestic refrigerator based on 0.2, 0.4, and 0.6 g/L TiO_2_ mixed with (Capella D) oil as an alternative to R134a and liquefied petroleum gas was charged as a refrigerant. The results showed that the cooling capacity and COP were higher than R134a by 18.74–32.72 and 10.15–61.49%, respectively. Additionally, the power consumed was lower than R134a by approximately 3.20–18.1. Karthick et al.^15^ studied the effect of nanolubricants on the performance of a refrigeration system based on the following samples: sample 1 (mineral oil + 0.02 vol% Al_2_O_3_ + 0.01 vol% TiO_2_), sample 2 (mineral oil + 0.01 vol% Al_2_O_3_ + 0.005 vol% TiO_2_), sample 3 (mineral oil + 0.05 vol% Al_2_O_3_), and sample 4 (mineral oil + 0.02 vol% Al_2_O_3_ + 0.02 vol% ZnO). R600a was used as a refrigerant. The results showed that COP was enhanced by 14.61%. All nanolubricants have the ability to improve the COP and save power consumption. Adelekan et al.^16^ studied the effect of nanolubricants on the performance of a domestic refrigerator based on 0.2 g/L, 0.4 g/L, and 0.6 g/L TiO_2_, and the liquefied petroleum gas was used as a refrigerant. The results indicated that nanolubricants achieved a reduction in power consumption by 14%, 9%, and 8% respectively. Subhedar et al.^17^ studied the effect of nanolubricants on the performance of refrigeration system based on 0.05 vol%, 0.075 vol%, 0.1 vol%, and 0.2 vol% of Al_2_O_3_ added to mineral oil, and R134a was used as a refrigerant. The results showed that 0.075 vol% achieved the best improvement in COP of approximately 85%, and saved approximately 27% compressor power. Additionally, 0.075 vol% was reported to be the best concentration of refrigeration system. Babarinde et al.^18^ studied the effect of nanolubricants on the performance of a refrigerator based on 0.4 and 0.6 g/L TiO_2_ added to mineral oil and R600a was charged as a refrigerant as an alternative to R134a. The results showed that 0.4 g/L TiO_2_ achieved the maximum value of COP and the minimum value of the power consumption. Selimefendigil and Bingölbalı^19^ studied the effect of nanolubricants on the performance of a vapor compression refrigeration system based on 0.5 vol%, 0.8 vol%, and 1 vol% TiO_2_ added to polyethylene glycol, and R134a was charged as a refrigerant. The results showed that 0.5 vol%, 0.8 vol%, and 1 vol% achieved improvements in COP of approximately 1.43%, 15.72%, and 21.42%, respectively; 1 vol% saved energy consumption by 15%. Sundararaj and Manivannan^20^ studied the effect of nanolubricants on the performance of a vapor compression refrigeration system based on 0.1 vol% Au, 0.2 vol% Au, 0.1 vol% HAuCl_4_, 0.2 vol% HAuCl_4_, 0.1 vol% Au and 0.05 vol% CNT, 0.2 vol% Au and 0.02 vol% of CNT mixed with polyethylene glycol oil, and R134a was charged as a refrigerant. The results showed that 0.2 vol% Au and 0.02 vol% CNT achieved the lowest power consumption compared to other compositions, the highest cooling capacity, and the best value of COP. Peyyala et al.^21^ studied the effect of nanolubricants on the performance of a vapor compression refrigeration system based on 0.1 vol% to 0.2 vol% Al_2_O_3_ mixed with mineral oil, and R410a was charged as a refrigerant. The results showed that the values of COP increase with increasing nanoparticle concentrations. Babarinde et al.^22^ studied the effect of nanolubricants on the performance of a vapor compression refrigeration system based on 0.2, 0.4, and 0.6 g/L graphene mixed with mineral oil, and R600a was charged as a refrigerant. The results showed that the nanolubricants exhibited the lowest power consumption, and the highest COP. Adelekan et al.^23^ studied the effect of nanolubricants on the performance of a domestic refrigerator based on 0.1 g/L, 0.3 g/L, and 0.5 g/L TiO_2_, mixed with mineral oil, and R600a was charged as a refrigerant The results indicated that nanolubricants exhibited the maximum values of COP and cooling capacity which were 4.99 and 290.83 kJ/kg respectively. Ajayi et al.^24^ studied the effect of nanolubricants on the performance of a vapor compression refrigeration system based on 0.5 g/l Al_2_O_3_ added to (Capella D) oil, and 100 g R134a was charged as a refrigerant. The results indicated that the nanolubricant achieved improvements in both the cooling capacity, and COP, and saved energy consumption. Senthilkumar and Anderson^25^ studied the effect of nanolubricants on the performance of a vapor compression refrigeration system, based on 0.2 g/L, 0.4 g/L, and 0.6 g/L SiO_2_, mixed with polyolester oil, and R410A was charged as a refrigerant. The results showed that 0.4 g/L SiO_2_ improved both the cooling capacity and COP and saved energy consumption. Senthilkumar et al.^26^ studied the effect of nanolubricants on the performance of a vapor compression refrigeration system based on 0.4 g/L and 0.6 g/L Al_2_O_3_/SiO_2_, and 40 and 60 g of R600a were used as refrigerants. The results showed that 0.6 g/L and 60 g of R600a achieved maximum cooling capacity, maximum COP, and minimum compressor work.

#### Evaluation of the refrigeration system based on nanorefrigerant

Pawale et al.^27^ studied the effect of a nanorefrigerant on the performance of a vapor compression refrigeration system based on 0.5 wt%, and 0.1 wt% Al_2_O_3_, dispersed into R134a. The results indicated that 0.5 wt% improved the performance of the system; however, the increase in the nanoparticle concentration caused a reduction in the performance of the system. Kumar et al.^28^ studied the effect of a nanorefrigerant on the performance of a vapor compression refrigeration system based on (1 g of ZnO/1 g SiO_2_), (1.5 g of ZnO/0.5 g of SiO_2_), and (0.5 g of ZnO/1.5 g of SiO_2_) dispersed into 0.5 kg R134a.The results indicated that COP improved approximately 26%. Manikanden and Avinash^29^ studied the effect of a nanorefrigerant on the performance of domestic refrigerators based on CuO, pure nano-Cuo, and Ag-doped nano-CuO dispersed into R290. The results showed that Ag-doped nano-CuO achieved the best performance compared to pure nano-CuO. The COP of Ag-doped nano-CuO improved approximately 29%, while the power consumed was reduced approximately 28%. Kundan and Singh^30^ studied the effect of a nanorefrigerant on the performance of a vapor compression refrigeration system based on 0.5 to 1 wt% Al_2_O_3_ dispersed into R134a.The results indicated that 6.5 L/h and 11 L/h volume flow rates of refrigerants achieved enhancements in the COP from 7.20 to 16.34% respectively at 0.5 wt% Al_2_O_3_. However, 1 wt% Al_2_O_3_ caused a reduction in COP at the same volume flow rates. Nagaraju and Reddy^31^ studied the effect of a nanorefrigerant on the performance of a vapor compression refrigeration system based on 0.05 to 0.8 wt% CuO dispersed into R134a.The results indicated that 0.8 wt% Cuo was the optimal concentration that achieved the maximum heat transfer enhancement, improved COP, and reduced the power consumed. Kumar and Tiwari^32^ studied the effect of a nanorefrigerant on the performance of a vapor compression refrigeration system based on R134a/Cu, R600a/Cu. The results indicated that R600a achieved the improvements in both the COP and cooling capacity of approximately 27.12% and 25% respectively, while the power consumed was less than that of R134a. Moreover dispersing 0.5 wt%, 1 wt%, and 1.5 wt% Cu into R600a improved the COP and cooling capacity compared to pure R600a, and reduced the power consumption. Kumar et al.^33^ studied the effect of a nanorefrigerant on the performance of vapor compression refrigeration system based on 0.01 vol% and 0.06 vol% ZrO_2_ dispersed into both R134a and R152a. The results showed that the COP improved by 33.45% based on (0.06 vol% of ZrO_2_-R152a). R152a when used as a refrigerant showed distinct environmental properties including zero ozone depletion potential and very low global worming potential. Mahdi et al.^34^ studied the effect of a nanorefrigerant on the performance of a vapor compression refrigeration system based on 0.01 vol% and 0.02 vol% Al_2_O_3_ dispersed into R134a. The results indicated that increasing the nanoparticle concentration improved COP by 3.33% to 12%, and reduced the power consumed by nearly 1.6% and 3.3%, respectively. Pandey and Singh^35^ studied the effect of a nanorefrigerant on the performance of a vapor compression refrigeration system based on 0.2, 0.4, and 0.6 vol% TiO_2_ dispersed into R134a.The results indicated that the COP improved approximately 11.1%. Additionally an increase or decrease in power consumption has not been observed, which indicates complete dissolution of the nanoparticles in the refrigerant.

#### Evaluation of the thermophysical properties of refrigerant and lubricant oil

Kedzierski et al.^36^ studied the thermophysical properties of nanolubricants based on Al_2_O_3_ and ZnO added to polyolester oil at temperatures ranging from 288 to 318 K. The results indicated that by increasing the nanoparticle concentration, the viscosity, density, and thermal conductivity increase, while increasing the temperature leads to a decrease in the viscosity and density. Sanukrishna and PrakashPrakash^37^ studied the thermal conductivity and viscosity of nanolubricants based on 0.07 to 0.8 vol% TiO_2_ mixed with poly-alkylene glycol with temperatures ranging from 20 °C to 90 °C. They found that by increasing the nanoparticle concentration all these parameters increase, while increasing the temperature leads to a decrease in these parameters. Zawawi et al.^38^ studied the thermal conductivity and viscosity of nanolubricants based on 0.02 to 0.1 vol% Al_2_O_3_/SiO_2_, Al_2_O_3_/TiO_2_, and TiO_2_/SiO_2_ mixed with poly-alkylene glycol oil at temperatures ranging from 303 to 353 K. The results indicated that 0.1 vol% Al_2_O_3_/TiO_2_/PAG improved the viscosity by approximately 20.50% at 303 K. While 0.1 vol% Al_2_O_3_/SiO_2_/PAG improved the thermal conductivity approximately 2.41% at 303 K. Harichandran et al.^39^ evaluated the density and kinematic viscosity of nanolubricants based on 0.1 to 0.4 vol% h-BN nanoparticles. The results showed that by increasing the nanoparticle concentration the density increased. Additionally the kinematic viscosity of pure oil and nanolubricants decreases with increasing temperatures and the kinematic viscosity of 0.4 vol% h-BN was approximately 14% higher than that of pure oil. Karthick et al.^15^ studied the thermal conductivity of the following samples, sample1 (mineral oil + 0.02 vol% Al_2_O_3_ + 0.01 vol% TiO_2_), sample 2 (mineral oil + 0.01 vol% Al_2_O_3_ + 0.005 vol% TiO_2_), sample 3 (mineral oil + 0.05 vol% Al_2_O_3_),and sample 4 (mineral oil + 0.02 vol% Al_2_O_3_ + 0.02 vol% ZnO). The results indicated that the nanolubricant based on 0.05 vol% Al_2_O_3_ achieved a higher value of thermal conductivity, while nanolubricant based on 0.01 vol% Al_2_O_3_ and 0.005 vol% TiO_2_ achieved the lowest value of thermal conductivity. Kumar et al.^40^ studied the viscosity of nanolubricants based on 0.2 to 1.0 wt% CuO. The results indicated that 0.2–1.0 wt% CuO improved the viscosity by approximately 17%, while, the viscosity decreased with increasing temperature. Jatinder et al.^41^ studied the thermal conductivity and viscosity of nanolubricants based on 0.1 to 0.6 g/L TiO_2_.The results indicated that the thermal conductivities were approximately 14.37–41.25% higher than that of pure lubricant, while the viscosity was approximately 2–6%. Moreover, the viscosity of nanolubricants decreases with increasing the nanoparticle concentration to 0.2 g/L and then increases with increasing concentration to reach the peak value at 0.6 g/L TiO_2_.

The addition of nanoparticles whether metal, metal oxide, or hybrid, to the refrigeration system, improves the performance of the system by improving the thermophysical properties of the base fluid. However the actual use of nanoparticles has not succeeded due to the high costs and instability of nanoparticles for a long time during their rotation within the refrigeration cycle. The use of nanoparticles as hybrids in recent years has attracted the interest of many researchers as an attempt to improve both the thermal properties and stability of nanoparticles.

**Figure 1:**
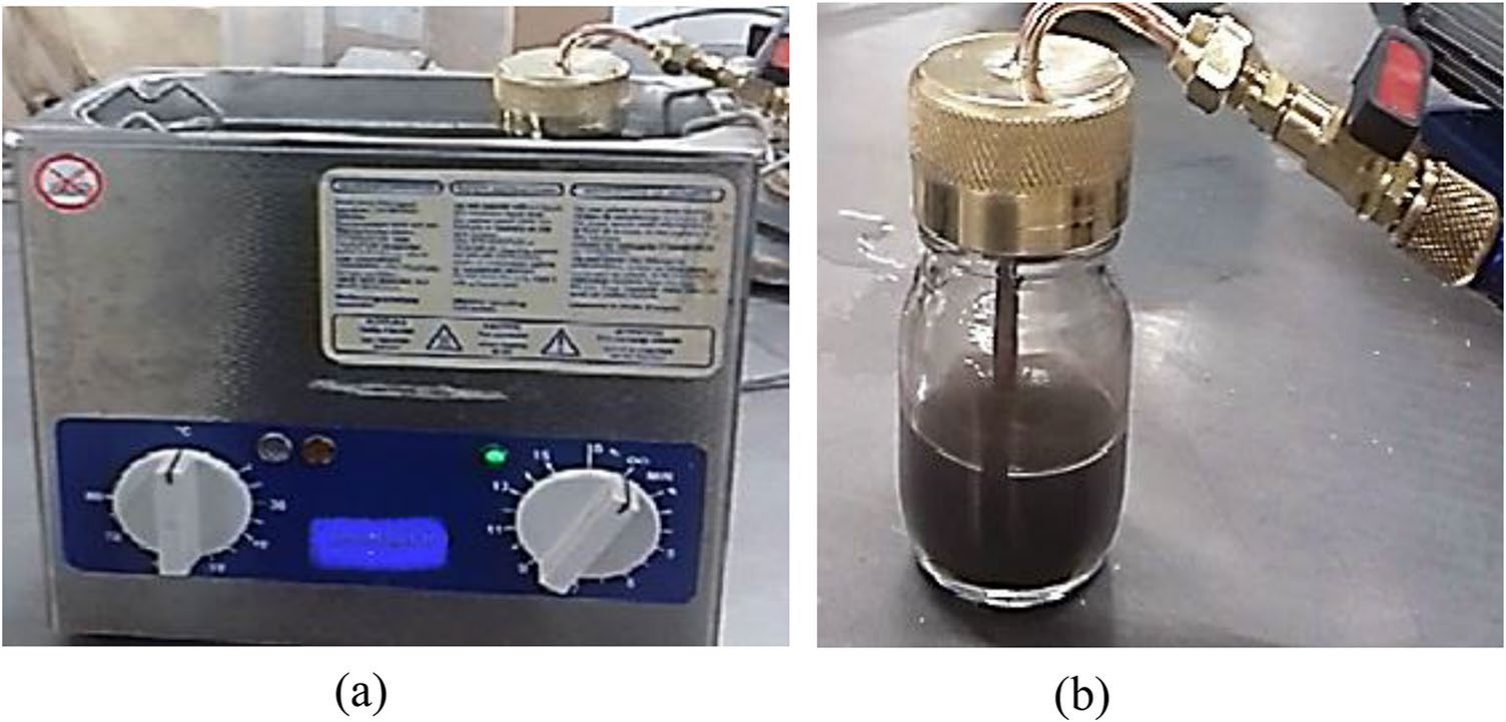
(**a**) Ultrasonic device, and (**b**) the mixing vessel of the nanorefrigerant.

In this research the nanoparticles were prepared as follows CuO, CeO_2,_ the first mixture consisted of 50% CuO with 50% CeO_2,_ the second mixture consisted of 60% CuO with 40% CeO_2_, the third mixture consisted of 70% CuO with 30% CeO_2_, the fourth mixture consisted of 40% CuO with 60% CeO_2_, and the fifth mixture consisted of 30% CuO with 70% CeO_2_. To investigate its ability to enhance the COP of the refrigeration system as well as improve the stability of the nanoparticles, a sample of the first mixture_,_ was weighed using a sensitive balance and then placed in the mixing container that was closed tightly and evacuated from the air using a vacuum pump. The container was weighed again to ensure that there was no loss in the amount of nanoparticles after the vacuum process. The mixing container was made from Pyrex. The following parts were installed with the container: cover made from copper with inner diameter 4 cm, copper tube L shaped, its length 6.5 cm inside the container is welded with a cap and its length outside the container 2.5 cm with an additional length up to 4 cm to place the valve of transfer the gas. This container was tested in terms of its resistance to leaks*.* Figure 1 shows the mixing container of this study. To achieve a stable solution of nanorefrigerant, the sample was exposed to an ultrasonic device, which works with a power of 320 W and a frequency of 35 kHz. This study achieved success in obtaining a homogeneous mixture for a whole day while the mixing process continued for one hour, which gave an indication that the use of nanoparticles as a mixture may help to obtain a more homogeneous and stable mixture for a longer time, which makes us start a series of studies in this field soon, Fortunately, the method of preparation used in this research, as the materials from which nanoparticles are prepared can be commonly found in all chemistry laboratories. Therefore, the cost barrier, which is one of the major obstacles to using nanoparticles, is broken. Thus, this research presents an attempt to break the barrier of high nanoparticle costs and obtain nanoparticles with high thermal conductivity at a reasonably affordable cost. In addition to presenting an alternative to hybrid nanoparticles, which is the mixture that may succeed in solving the problem of nanoparticle stability for a long time?

## Materials and methods

This section includes four parts, the first part in which an experiment was performed on a vapor compression refrigeration system using R134a as a refrigerant, and the coefficient of performance was calculated based on the change in the enthalpy of the refrigerant. Then Ansys Fluent software version 19.0 was used to calculate the coefficient of performance theoretically to make a comparative study between the experimental and theoretical results. In the second part seven types of nanoparticles were prepared, and the preparation process will be explained in detail later. In the third part nanoparticles are added to the refrigeration system theoretically to verify their effect on the coefficient of performance of the refrigeration system. In the fourth part some numerical equations available in the literature are used to calculate the thermophysical properties of R134a using different nanoparticle concentrations.

### Experimental work

The vapor compression refrigeration system must be evacuated to remove substances such as air, water, moisture and inert gases from the refrigeration system which causes various effects that lead to a reduction in of the life of the cycle. Using the air compressor, the air is pumped, the pressure mater is monitored, and the leak test is performed to ensure that there is no leakage during the operation of the system.

An experiment was carried out in a laboratory under normal conditions at Yildiz Technical University in Istanbul, Turkey, The system was charged using R134a as a working fluid. The selection of R134a as a refrigerant for the refrigeration system was based on the fact that, it is suitable for all types of oxides, it is safe during the operation process, it has zero ozone depletion potential, it is also non-flammable, and inexpensive, and most of the previous studies used it in refrigeration systems. Digital meters were used to monitor the temperatures and pressures at the inlets and exits of a compressor, a condenser, and an evaporator, A digital wattmeter was used to monitor power consumption and a digital flow meter was used to monitor the mass flow rate of R134a, negligible heat loss to the surroundings and the changes in the kinetic and potential energy.

Every experiment was performed several times and waiting lasted 15 min before taking the reading from pressure and temperature meters to obtain the highest accuracy, and steady-state performance.

**Figure 2:**
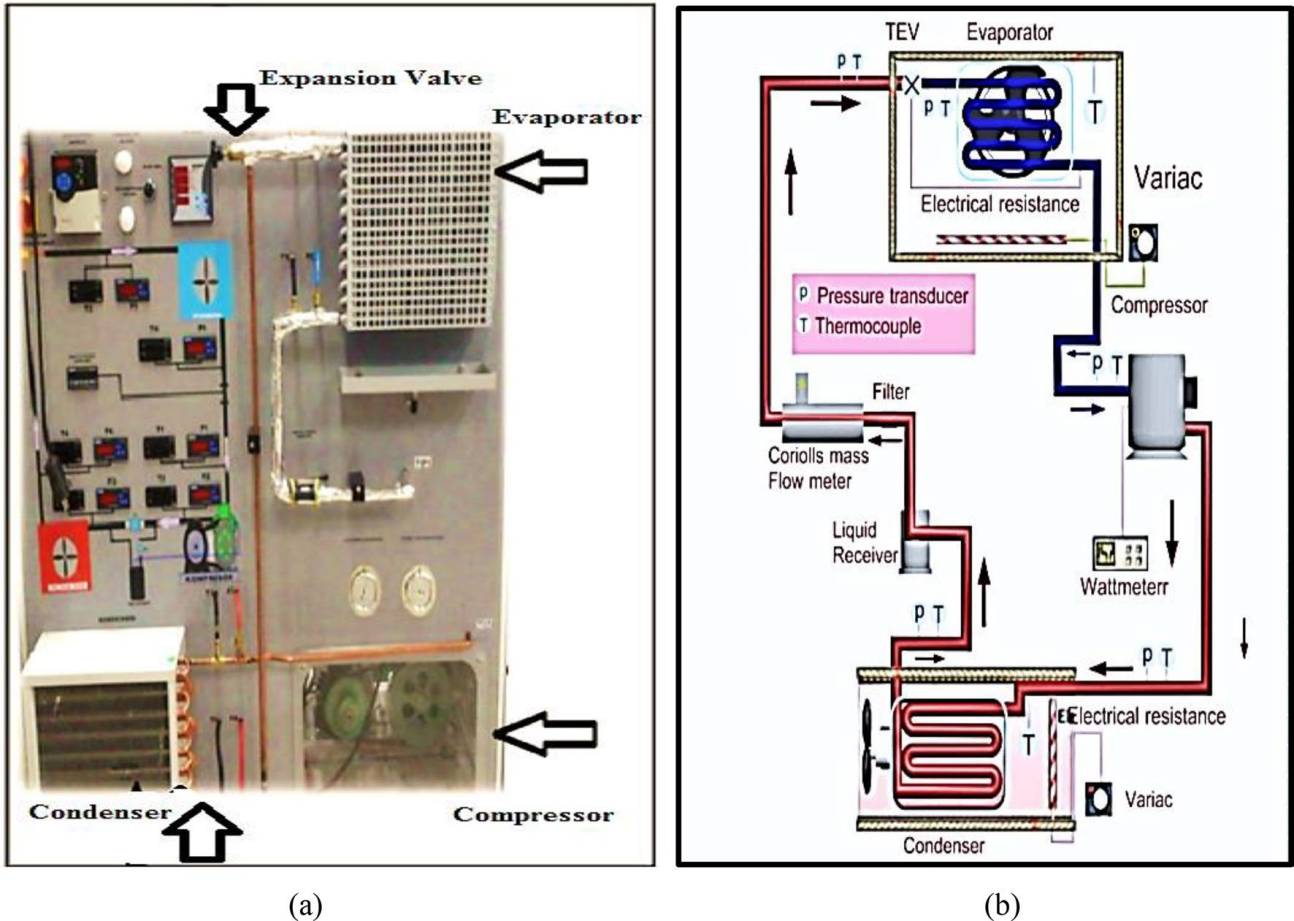
(**a**) Experimental work and its corresponding parameters and (**b**) schematic diagram of the experiment drawn by Mechanical Engineer Taher Abdaladeem Jaber.

Table 1 shows the technical details of the experimental system. An experimental setup and its schematic diagram are presented in Fig. 2. The experiment consists of a compressor, condenser, evaporator, and expansion valve.

**Table 1:** Technical details of the experimental system.

No	Components	Characteristics	No	Components	Characteristics
1	Compressor	Bitzer compressor 1/2 HP	6	Suction line	3/8-inch copper pipe
2	Condenser	Air cooled condenser 1/2 HP	7	Discharge line	1/4-inch copper pipe
3	Evaporator	Emersion coil 1/3 HP	8	Frequency rating	51 Hz
4	Expansion valve	Automatic expansion valve	9	Defrost unit	Automatic
5	Voltage rating	220 V AC voltage supply	10	Door type	Single closed door

#### Experimental procedure

**Figure 3:**
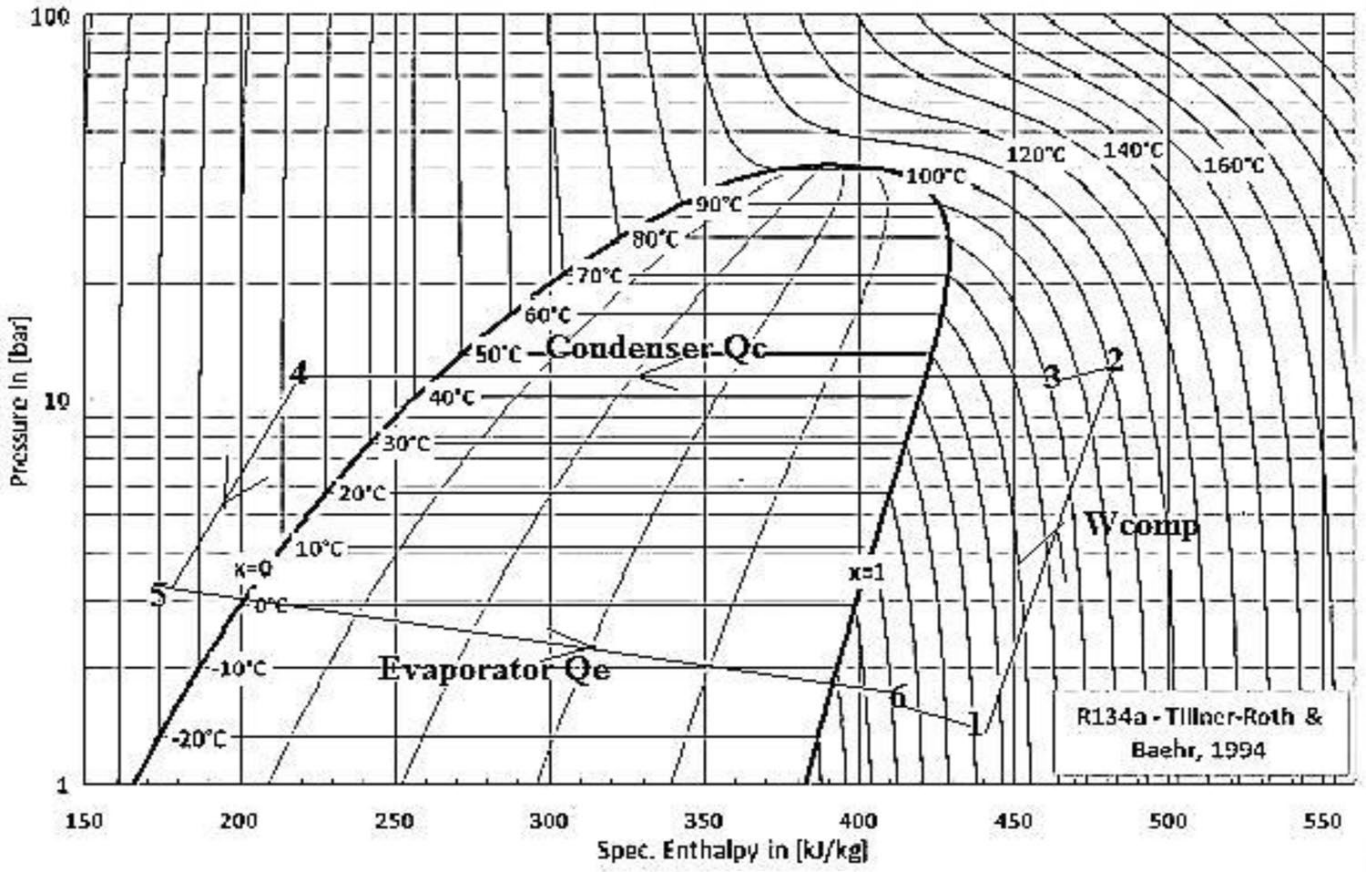
The cycle of the experiment on the P–h diagram.

The temperatures and pressures that are read from meters at the inlet and outlet of both the evaporator, condenser, and compressor are entered into the Engineering Equation Solver (EES). This software helps in determining the enthalpy of R134a and the change in the gas phase, where two phases of the gas cycle were recorded in the system namely the superheated vapor enthalpy (h1, h2) at the inlet and exit of the compressor, the superheated vapor enthalpy (h3) at the inlet of the condenser, the compressed liquid enthalpy (h4) at the exit of the condenser, the compressed liquid enthalpy (h5) at the inlet of the evaporator, and the superheated vapor enthalpy (h6) at the exit of the evaporator. Listed governing equations have been employed for analysis^20^. Characteristics R134a utilized in the experimental setup are given in Table 2 and the cycle of the experiment on the P–h diagram is presented in Fig. 3.

1 $$ {\dot{\text{W}}} = {\dot{\text{m}}}\,\left( {{\text{h}}2 - {\text{h}}1} \right) $$

2 $$ {\text{Q}}\,{\text{evaporator}} = {\dot{\text{m}}}\,({\text{h}}6 - {\text{h}}5) $$

3 $$ {\text{Q}}\,{\text{condenser}} = {\dot{\text{m}}}\,({\text{h}}3 - {\text{h}}4) $$

4 $$ {\text{COP}} = \frac{{\left( {{\text{h}}6 - {\text{ h}}5} \right)}}{{\left( {{\text{h}}2 - {\text{h}}1} \right)}} $$

**Table 2:** Characteristics of R134a^42^.

No	Characteristics		No	Characteristics	
1	Name	1,1,1,2 tetrafluoroethane	11	Saturated vapor pressure at 20 °C	774.3 kPa
2	Chemical formula	CF3CFH2	12	Latent heat of vaporization	198.6 kJ/kg
3	Molecular weight	102.03 g/mol	13	Liquid density	1294.8 kg/m^3^
4	Composition	pure	14	Vapor density	14.43 kg/m^3^
5	ASHRAE safety classification	A1	15	Liquid Cp	1.341 kJ/kg °C
6	Ozone Depletion Potential	zero	16	Vapor Cp	0.90 kJ/kg °C
7	Lifetime in the atmosphere	13	17	Liquid thermal conductivity at 25 °C	0.0824 W/m K
8	Critical temperature	101.1 °C	18	Vapor thermal conductivity at 25 °C	0.0145 W/m K
9	Critical pressure	4.06.3 MPa	19	Liquid viscosity at 25 °C	0.202 mPa s
10	Normal Boiling Point NBP	− 26.4 °C	20	Vapor viscosity at 25 °C	0.012 mPa s

#### Numerical method, geometry and mesh of (VCRS)

Both the evaporator and the condenser were chosen to study the effect of the average temperature on the performance of the refrigeration system. Achieving the mathematical model requires the following stages (1) The geometry of each evaporator and condenser was designed using Solid Works software based on the real dimensions that are shown in Table 3, where this software helped to draw quickly and accurately and then save as the Para Solid format to import the file to Ansys Fluent for analysis. (2) Meshing plays a vital role in achieving accuracy. A high quality simulation is required for successful numerical simulation for this reason mesh independency analysis is performed for many cases with various numbers of elements and nodes to check the validity of the quality of the mesh on the solution. The mesh statistics used the total number of elements 2,809,136 and total numbers of nodes 2,971,904, these results of the mesh independency analysis are presented in Table 4.

**Table 3:** Dimensions and characteristics of both the condenser and evaporator.

No	Condenser measurements	Dimensions	No	Evaporator measurements	Dimensions
1	Length	35 cm	1	Length	33 cm
2	Width	17 cm	2	Width	10 cm
3	Height	28 cm	3	Height	28 cm
4	Diameter of the copper tube	0.25 inch	4	Diameter of copper tube	0.25 inch
5	Number of twists and copper tube	18	5	Number of twists and copper tube	18
6	Distance between one tube and another	5 cm	6	Area of evaporator	0.12 cm^2^
7	Area of condenser	0.177 cm^2^	7	Number of tubes	18
8	length of the tube inside the condenser	7.2 m	8	Fan speed	1300 rpm
9	The thickness of aluminum plate	0.3 mm	9	Air velocity	638 m/s
10	Number of plates	600	10	Evaporator type	emersion coil
11	Single plate dimensions	35*17*34 cm	11	Distance between one tube and another	3 cm
12	Fan speed	1300 rpm	12	length of the tube inside the evaporator	6.48 m
13	Air velocity	638 m/s			
14	Condenser type	Air cooler

**Table 4:** Number of elements and nodes in four different levels of mesh independency analysis.

Case	Element	Node	Pressure (Pa)
1	634,783	823,404	6.29
2	1,323,465	1,534,078	7.23
3	2,026,567	2,102,577	7.6
4	2,809,136	2,971,904	7.61

**Figure 4:**
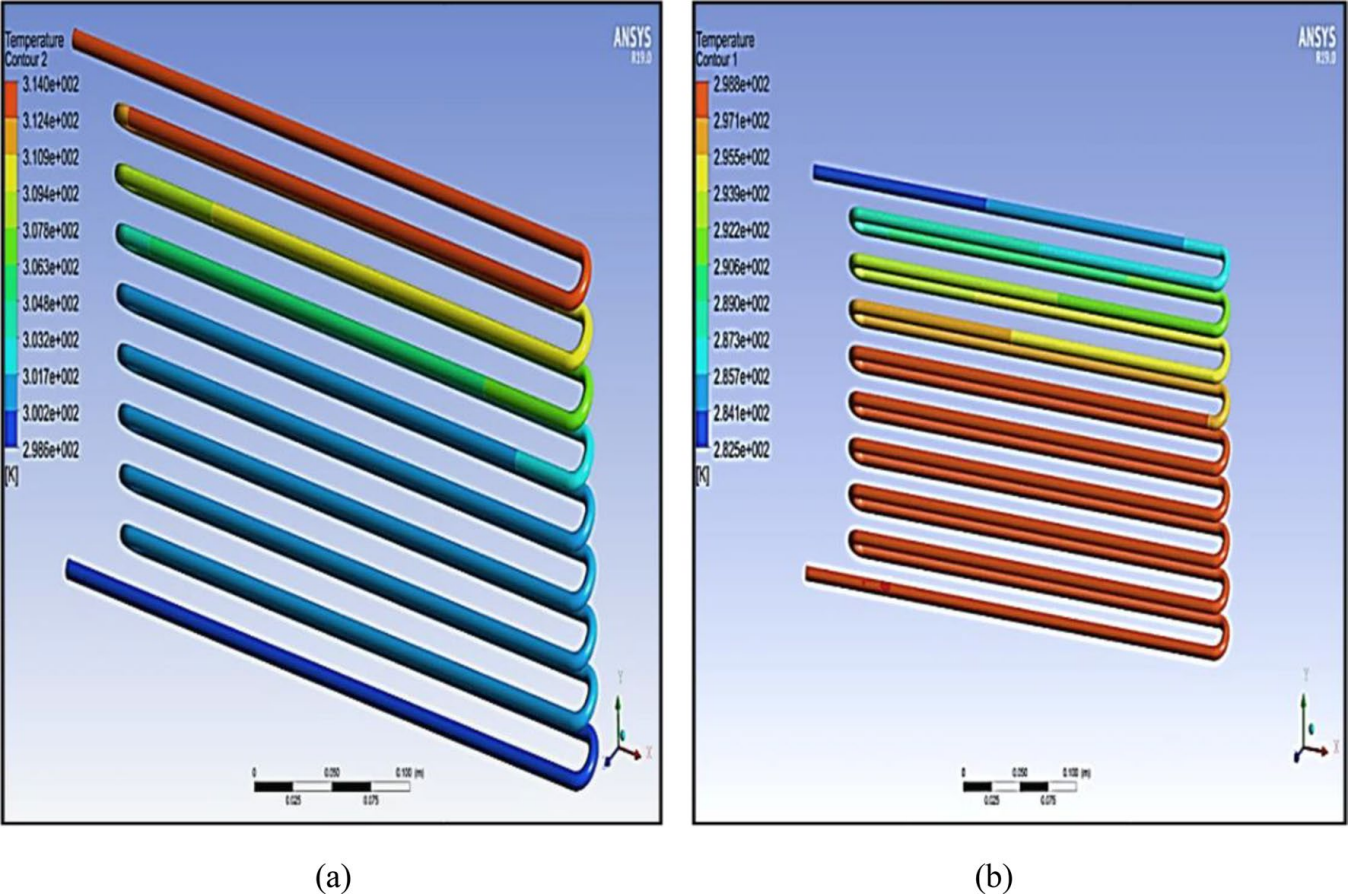
(**a**) The thermal gradient of the condenser and (**b**) the thermal gradient of the of the evaporator.

Several contours have been obtained to show the gradient in the temperature, pressure, and velocity inside both the condenser and the evaporator, and some of these contours are presented in Fig. 4. These contours determine the velocity, pressure, and temperature of the refrigerant (R134a) as it circulates through the tubes. The colors shown in the drawing indicate that the red colors give the highest reading, the blue colors the lowest reading and the colors between red and blue are between the highest and the lowest in all cases of gradation, whether it is temperature, pressure or velocity.

Step 3) Fluent setup after the geometry and mesh the equations used in the solution are entered, which will be as follows: the continuity, momentum, and energy equations, where these equations extract all the theoretical values represented in the amounts of heat absorbed inside the evaporator and rejected from the condenser, and the energy consumed from the compressor, as well as the theoretical temperatures and pressures to be compared with the experimental values, thus moving to the solution stage directly and from there to the results obtained.

### Preparation of nanoparticles

The nanoparticles were prepared based on nitrates, distilled water, and ammonia. The preparation process was briefed as follows:

1. The temperature was raise to 80 °C for 1 h while maintaining the mixing speed at 375 rpm
2. Ammonia Is added while maintaining the temperature at 60 °C, mixing speed at 375 rpm, and pH = 10 ± 1
3. Increase the temperature until 90 °C where the copper oxide is deposited
4. Reduce the temperature of the solution to room temperature
5. Filtration process
6. Drying using an electric oven at 110 °C
7. Milling process
8. Screening process; and
9. Packing. According to the equations below, the nanoparticles were prepared as indicated clearly in Table 5 and their physical and chemical properties are shown in Table 6.
  5 $$ M = \frac{W}{{M_{W}^{{}} }} $$
  6 $$ C = \frac{n}{v} $$
  where *M*: Mole (g/mol), *W*: Weight (g), *Mw*: Molecular weight (mol), *C*: Mole concentration (mol/L), *n*: Number of moles.

**Table 5:** The quantities obtained in grams from the mixtures.

Mixture number	Percentages		Molar concentration		Molar concentration of the mixtures (m/l)	The quantity (g)
	CuO (%)	CeO_2_ (%)	Cu (NO3)2 3H_2_O (m/l)	Ce (NO_3_)3 6H_2_O (m/l)		
1	50	50	0.20867	0.09673	0.1527	12.67
2	60	40	0.250408	0.07753	0.181258	11.44
3	70	30	0.300075	0.05834	0.21530	10.97
4	40	60	0.16694	0.11622	0.13651	13.07
5	30	70	0.125687	0.135569	0.13260	13.85

**Table 6:** Physical and chemical properties of nanoparticles^43^.

No	Nanoparticles	Molar mass (g/mol)	Density (kg/m^3^)	Appearance	Thermal conductivity (W/m K)	specific heat (J/kg K)
1	CuO	79.55	6320	Black to brown powder	32.9	536
2	CeO_2_	172.115	6100	White or pale yellow	11.715	352
3	50%CuO + 50%CeO_2_	125.833	6210	Black to brown powder	22.31	444
4	60%CuO + 40%CeO_2_	116.576	6232	Black to brown powder	24.43	462.4
5	70%CuO + 30%CeO_2_	107.32	6254	Black to brown powder	26.54	480.8
6	40%CuO + 60%CeO_2_	135.089	6188	Black to brown powder	20.19	425.6
7	30%CuO + 70%CeO_2_	144.346	6166	Black to brown powder	18.07	407.2

Table 5 shows that the weights of substances involved in the reaction were converted to moles by dividing the weight by the molecular weight as indicated in Eq. (5). Then the molar concentration was calculated as indicated in Eq. (6) by dividing the number of moles by the volume of the solvent in liters.

Note that: the molecular weights of the reactants were as follows

$$ \begin{aligned} & {\text{Cu}}\left( {{\text{NO}}_{3} } \right)_{2} 3{\text{H}}_{2} {\text{O}} = 241.606 \\ & {\text{Ce}}\left( {{\text{NO}}_{3} } \right)_{3} 6{\text{H}}_{2} {\text{O}}\,{\text{Ce}} = 434.22 \\ & {\text{CuO}} = 79.5 \\ & {\text{CeO}}_{2} = 172.1 \\ \end{aligned} $$

### Computational fluid dynamics (CFD)

After completing the geometry and mesh stages referred to in “Numerical method, geometry and mesh of (VCRS)” section, the refrigeration system for this study is ready to receive the nanoparticles. This stage is called the setup where the type of solver is chosen as pressure based the velocity formulation is absolute, the time is taken as steady and the gravity is taking into account as − 9.81 m/s^.^ This study included two models the first is the viscous model standard k – epsilon and the second is the mixture model which is the most common model used to simulate the flow of more than one phase in (CFD). To make the phase setup, the properties of both R134a are entered into the liquid and gaseous phases, and the nanoparticles with the proportions specified for it depend on the nanofluid equations and deal with nanoparticles and R134a based on becoming one homogeneous material as shown below.

7 $$\mathrm{Keff}=\mathrm{kbf }\left(\frac{\mathrm{kp}+2\mathrm{kbf}+2\left(\mathrm{kp}-\mathrm{kbf}\right){\upvarphi }}{\mathrm{kp}+2\mathrm{kbf}-\left(\mathrm{kp}-\mathrm{kbf}\right){\upvarphi }}\right)$$

where Keff, Kbf, and KP are the thermal conductivities of the nanorefrigerant, base refrigerant in the liquid phase, and particle respectively and ϕ is the particle volume fraction

8 $${\upmu }_{\mathrm{nr}}={\upmu }_{\mathrm{r}}\frac{1}{{(1-{\varnothing })}^{2.5}}$$

where ${\upmu }_{\mathrm{nr}}$ and, ${\upmu }_{\mathrm{r}}$ are the dynamic viscosities of the nanorefrigerant and refrigerant respectively.

The density and specific heat of the nanorefrigerant are shown in Eqs. (9) and (10)

9 $${\uprho }_{\mathrm{eff}=\left(1-{\varnothing }\right){\uprho }_{\mathrm{f}+ }{\varnothing }{\uprho }_{\mathrm{np}}}$$

10 $${C}_{pnf}=\frac{\left(1-\varnothing \right)\left(\rho cp\right) Bf+ \varnothing \left(\rho cp\right)NP}{\left(1-\varnothing \right)\rho BF+\varnothing \rho NP}$$

where ${\varnothing },\mathrm{ \rho bF},\mathrm{\rho nP}$,$\mathrm{and Cp are a}$ volume fraction of nanoparticles, density of base fluid, density of nanoparticles, specific heat of base refrigerant and specific heat of nanoparticles^44,45^.A mixture model solves the momentum, continuity, and energy equations for the mixture, and solves the equation of volume fraction for the secondary phases^44,45^ A continuity equation for the volume fraction of one (or more) of the phases. For the qth phase, this equation has the following form:

11 $$\frac{1}{{\uprho }_{\mathrm{q}}}[\frac{\partial }{\partial \mathrm{t}}\left({{\upalpha }}_{\mathrm{q}}{\uprho }_{\mathrm{q}}\right)+\nabla .\left({{\upalpha }}_{\mathrm{q}}{\uprho }_{\mathrm{q}}{{{\overset{\lower0.5em\hbox{$\smash{\scriptscriptstyle\rightharpoonup}$}} {\text{v}} }}}_{\mathrm{q}}\right)= {\mathrm{S}}_{{{\upalpha }}_{\mathrm{q}}}+\sum_{\uprho =1}^{\mathrm{n }}({{\dot{\mathrm{m}}}_{\uprho{\rm q}}-{\dot{\mathrm{m}}}_{\mathrm{qp}})}$$

where ${\dot{\mathrm{m}}}_{\uprho{\rm q}},\mathrm{ t}$ he mass is transfer from phase $\uprho$ to phase $\mathrm{q}$, ${\dot{\mathrm{m}}}_{\mathrm{qp}}$ the mass transfer from phase $\mathrm{q}$ to phase $\uprho$.

A single momentum equation is solved throughout the domain; it is dependent on the volume fractions of all phases through the properties $\mathrm{\rho and \mu }$.

12 $$\frac{\partial }{\partial \mathrm{t}}\left(\uprho {{\overset{\lower0.5em\hbox{$\smash{\scriptscriptstyle\rightharpoonup}$}} {\text{v}} }}\right)+\nabla .\left(\uprho  {{\overset{\lower0.5em\hbox{$\smash{\scriptscriptstyle\rightharpoonup}$}} {\text{v}} }} {{\overset{\lower0.5em\hbox{$\smash{\scriptscriptstyle\rightharpoonup}$}} {\text{v}} }} \right)=-\nabla\uprho +\nabla .[\upmu (\nabla {{\overset{\lower0.5em\hbox{$\smash{\scriptscriptstyle\rightharpoonup}$}} {\text{v}} }}+\nabla {{{\overset{\lower0.5em\hbox{$\smash{\scriptscriptstyle\rightharpoonup}$}} {\text{v}} }}}^{\mathrm{T }})\uprho {{\overset{\lower0.5em\hbox{$\smash{\scriptscriptstyle\rightharpoonup}$}} {\text{g}} }}+{{\overset{\lower0.5em\hbox{$\smash{\scriptscriptstyle\rightharpoonup}$}} {\text{F}} }}$$

The energy equation, is also shared among the phases

13 $$\frac{\partial }{\partial \mathrm{t}}\left(\mathrm{\rho E}\right)+\nabla .\left({{\overset{\lower0.5em\hbox{$\smash{\scriptscriptstyle\rightharpoonup}$}} {\text{v}} }}\left(\mathrm{\rho E}+\mathrm{P }\right)\right)= \nabla .\left({\mathrm{k}}_{\mathrm{eff}}\nabla \mathrm{T}\right)+{\mathrm{S}}_{\mathrm{h}}$$

The amount of nanoparticles that was added to R134a was 2.6 g, while the amount of R134a was 1039 g, as this was the amount that the system was operating within the first part of the experiment to become a mass fraction of 0.25 wt%. The theoretical results obtained by adding a quantity of nanoparticles are presented and their effects on the performance of the refrigeration system are discussed in the discussion.

### Thermophysical properties of the nanorefrigerant

The variation of thermophysical properties of nanorefrigerants based on R134a consisting of different volume fractions of nanoparticles ranging between 0.05 and 0.33 vol% are indicated clearly in Tables 7, 8, 9 and 10, where the thermal conductivity of the nanorefrigerant was conducted by the Maxwell equation indicated in Eq. (7). The Brinkman model was chosen in this study to calculate the effect of different volume concentrations on the viscosity of the nanorefrigerant indicated in Eq. (8). The density and specific heat of the nanorefrigerant can be calculated using Eqs. (9) and (10).

**Table 7:** Theoretical thermal conductivity of the nanorefrigerant.

Theoretical thermal conductivity (W/m K)	Volume fraction $\varphi$						
	0.05	0.09	0.1	0.2	0.24	0.28	0.33
CuO	0.09577	0.10716	0.11016	0.14431	0.16046	0.17839	0.20377
CeO_2_	0.09558	0.10680	0.10976	0.14328	0.15909	0.17661	0.20136
50%CuO + 50%CeO_2_	0.09572	0.10706	0.11006	0.14404	0.16010	0.17792	0.20313
60%CuO + 40%CeO_2_	0.09573	0.10709	0.11009	0.14411	0.16020	0.17805	0.20331
70%CuO + 30%CeO_2_	0.09574	0.10711	0.11011	0.14417	0.16028	0.17815	0.20345
40%CuO + 60%CeO_2_	0.09570	0.10703	0.11002	0.14395	0.15998	0.17777	0.20293
30%CuO + 70%CeO_2_	0.09568	0.10699	0.10998	0.14384	0.15984	0.17758	0.20267

**Table 8:** Theoretical dynamic viscosity of the nanorefrigerant.

Theoretical dynamic viscosity (mPa/s)	Volume fraction $\varphi$						
	0.05	0.09	0.1	0.2	0.24	0.28	0.33
	0.22964	0.25571	0.26287	0.35288	0.40116	0.45922	0.54975

**Table 9:** Theoretical density of the nanorefrigerant.

Theoretical density (kg/m^3^)	Volume fraction $\varphi$						
	0.05	0.09	0.1	0.2	0.24	0.28	0.33
CuO	1546.06	1747.07	1797.32	2299.84	2500.85	2701.86	2953.12
CeO_2_	1535.06	1727.27	1775.32	2255.84	2448.05	2640.26	2880.52
50%CuO + 50%CeO_2_	1540.56	1737.17	1786.32	2277.84	2474.45	2671.06	2916.82
60%CuO + 40%CeO_2_	1541.66	1739.15	1788.52	2282.24	2479.73	2677.22	2924.08
70%CuO + 30%CeO_2_	1542.76	1741.13	1790.72	2286.64	2485.01	2683.38	2931.34
40%CuO + 60%CeO_2_	1539.46	1735.19	1784.12	2273.44	2469.17	2664.90	2909.56
30%CuO + 70%CeO_2_	1538.36	1733.21	1781.92	2269.04	2463.89	2658.74	2902.30

**Table 10:** Theoretical specific heat of the nanorefrigerant.

Theoretical specific heat	Volume fraction $\varphi$						
	0.05	0.09	0.1	0.2	0.24	0.28	0.33
CuO	1.1765	1.0789	1.0579	0.8986	0.8528	0.8138	0.7725
CeO_2_	1.1445	1.0267	1.0012	0.8061	0.7496	0.7018	0.6499
50%CuO + 50%CeO_2_	1.1602	1.0524	1.0286	0.8519	0.8007	0.7571	0.7108
60%CuO + 40%CeO_2_	1.1633	1.0575	1.0347	0.8609	0.8108	0.7681	0.7228
70%CuO + 30%CeO_2_	1.1667	1.0629	1.0406	0.8706	0.8216	0.7798	0.7355
40%CuO + 60%CeO_2_	1.1571	1.0473	1.0236	0.8429	0.7907	0.7461	0.6988
30%CuO + 70%CeO_2_	1.1538	1.0419	1.0178	0.8334	0.7800	0,7345	0.6862

## Results and discussion

This section includes four parts; the first part includes discussing the results obtained by conducting an experiment on the refrigeration system and comparing these results with a simulation that was done using Ansys fluent 19.0 software. The second part includes discussing the results obtained from the preparation of nanoparticles. The third part includes discussing the results obtained from the theoretical study where nanoparticles were introduced to the refrigeration system to investigate their effects on the coefficient of performance of the system and the fourth part includes discussing the results obtained from numerical equations available in the literature to calculate thermophysical properties of R134a using different nanoparticle concentrations.

### Comparison between the experimental and theoretical results of the VCRS

**Figure 5:**
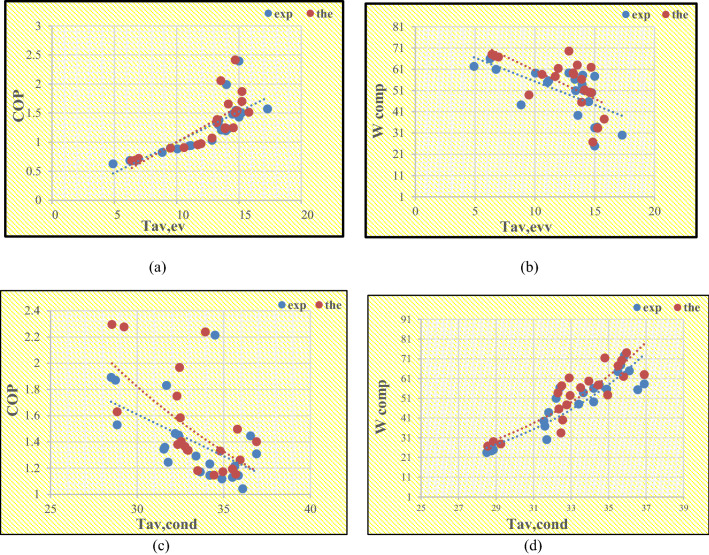
(**a**) The effect of Tav, ev on Cop, (**b**) The effect of Tav, ev on the power consumption, (**c**) The effect of Tav, cond on COP and (**d**) The effect of Tav, cond on the power consumption.

A theoretical model was designed for the evaporator and condenser with specifications similar to the experimental system; the results obtained were illustrated graphically to show the effects of the average temperature of both the evaporator and condenser on COP, and WC as shown in Fig. 5a–d. The COP at varying evaporator temperatures is presented in Fig. 5a; an increase in evaporator temperatures causes an increase in COP due to an increase in the refrigeration effect due to an increase in both the enthalpy difference and mass flow rate of R134a through the evaporator, and a decrease in compressor work. The power consumption at varying evaporator temperatures is presented in Fig. 5b; an increase in evaporator temperatures causes a decrease in power consumption, due to an increase in suction temperature, which causes an increase in both the vaporization pressure and density suction vapor entering the compressor, which leads to an increase in the mass flow rate of R134a through the compressor for a given piston displacement and decreases power consumption. The effects of the average temperature of the condenser on the COP are presented in Fig. 5c; it decreases as the condenser temperature increases due to a decrease in the refrigeration effect and an increase in compressor work, An increase in condenser temperatures causes an increase in the heat rejection, due to an increase in both the enthalpy difference and mass flow rate of R134a through the condenser. On the other hand, increasing the condenser temperature will cause an increase in power consumption as presented in Fig. 5d.

To achieve high accuracy, the experiment was divided into several cases, where each case included five experiments and each experiment was repeated three times. The experiment that gave the most convergence with the theoretical value calculated using Ansys fluent was chosen to then capture all these points to be plotted with the average temperatures of the evaporator and condenser. On critical examination of the results obtained from the experimental and simulation it has been observed that the results obtained from both methods are in good agreement with each other. This confirms that the results are identical and that there is no requirement for any correction factor, as shown by the results. In agreement with previous studies, our results confirm the increase in COP with the temperature of the evaporator and its decrease with the increase in the temperature of the condenser, as well as the decrease in the energy consumption with an increase in the average temperature of the evaporator and an increase with the increasing average temperature of the condenser.

### The results obtained from the preparation of nanoparticles

**Figure 6:**
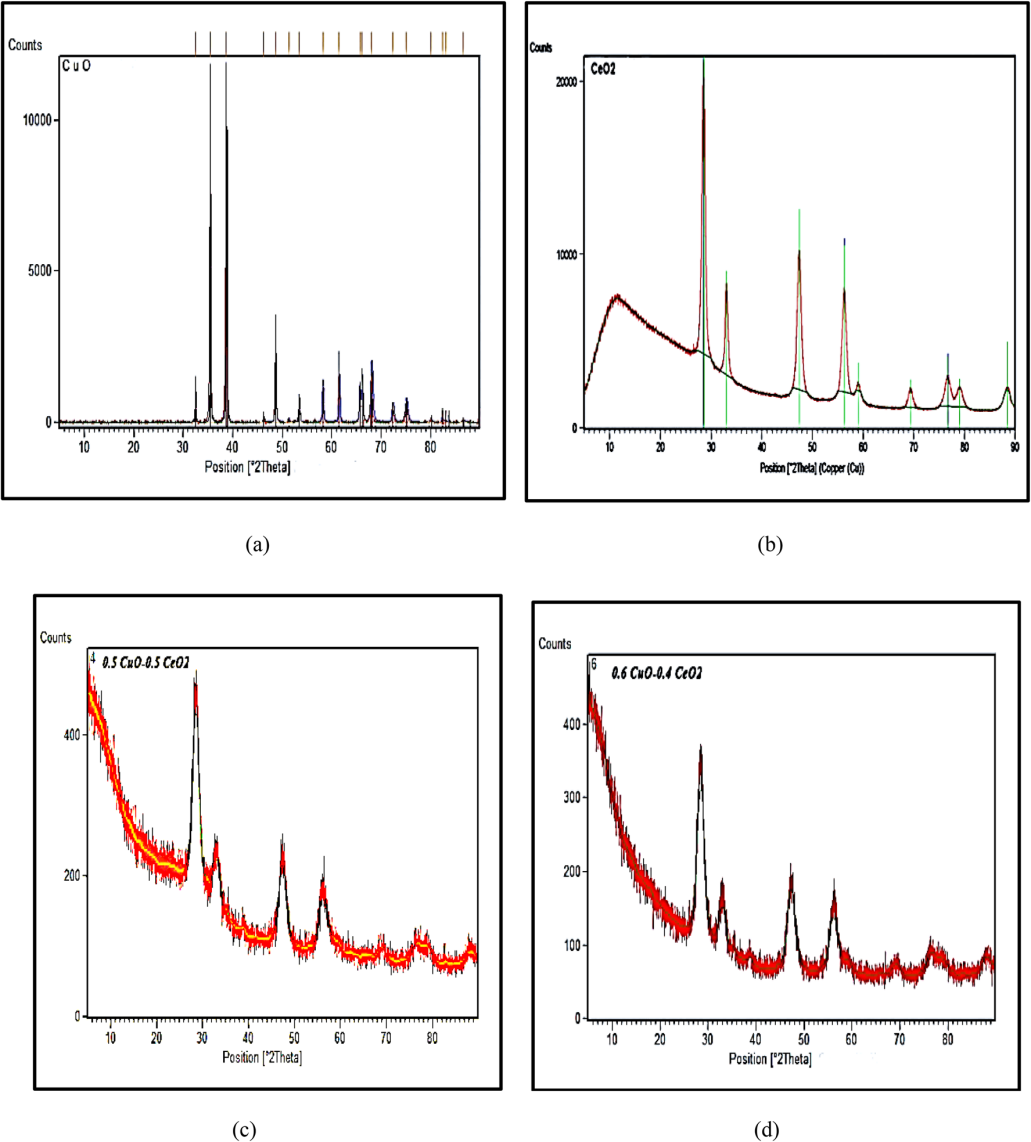
XRD Pattern of (**a**) Pure CuO, (**b**) Pure CeO_2_, (**c**) 0.5% CuO, 0.5% CeO_2_, (**d**) 0.6% CuO, 0.4% CeO_2_ Nanoparticles.

Nanoparticle characterization was carried out at Huazhong University of Science and Technology in China on September 24, 2019 by using XRD analysis and scanning electron microscopy (SEM) images. The nanoparticle preparation results are presented in Fig. 6a–d. The XRD pattern was scanned from 20 to 80 degrees and confirmed the nanocrystalline nature of CuO. All of the peaks agreed in position and intensity with the database standard (JCPDS 00-045-0937) of the face centered cubic CuO crystal with the fluorite structure. The absence of additional diffraction peaks confirms the nanocrystalline nature and purity of the samples.

The XRD pattern of CeO_2_ was scanned from 20 to 80 degrees and confirmed the nanocrystalline nature of CeO_2_. All of the peaks agreed in position and intensity with the database standard (JCPDS 00-004-0593) of the face centered cubic CeO_2_ crystal with the fluorite structure. The absence of additional diffraction peaks confirms the nanocrystalline nature and purity of the samples.

**Figure 7:**
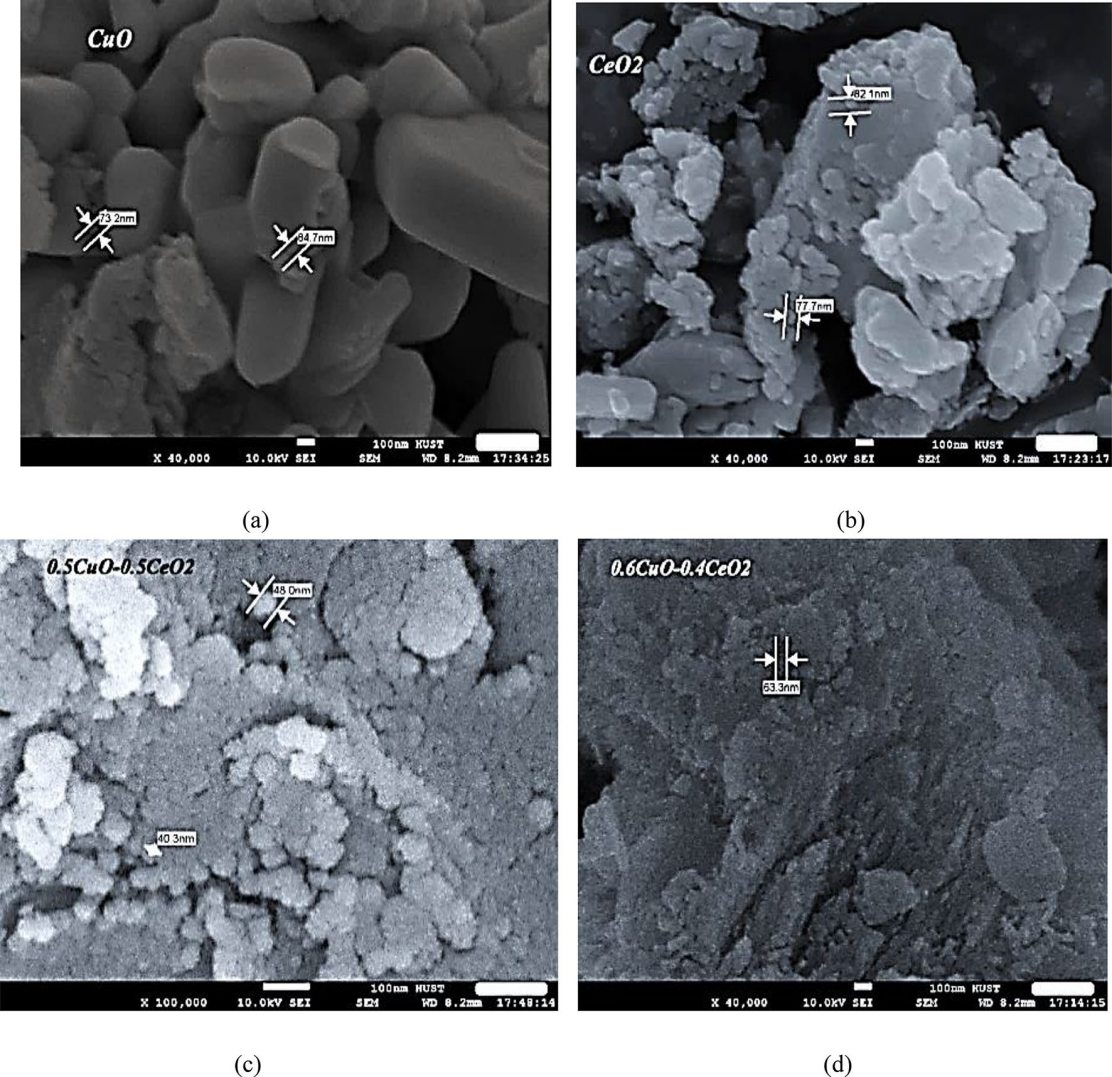
(**a**) Spheral CuO, (**b**) Spheral CeO_2_, (**c**) Spheral 0.5% CuO + 0.5% CeO_2_, (**d**) Spheral0.6% CuO + 0.4% CeO_2_.

The SEM images proved that the particles of the samples were approximately spherical in shape and the particle sizes of CuO, CeO_2_, 0.5%CuO + 0.5% CeO_2_, and 0.6% CuO + 0.4% CeO_2_ were observed to be 78.95 nm, 79.9 nm,44.15 nm, and 63.3 nm based on the SEM images respectively, as seen in Fig. 7a–d. This research succeeded in preparing nanoparticles with suitable diameters. Cerium oxide was used for the first time to determine its effect on the performance of the refrigeration system. It is expected that this study will open the door to future studies to reveal new properties of cerium oxide as a mixture with copper oxide; in particular, they were prepared by the same method as a homogeneous mixture that has the properties of both oxides.

### The results obtained from the theoretical addition of nanoparticles into the VCRS

**Figure 8:**
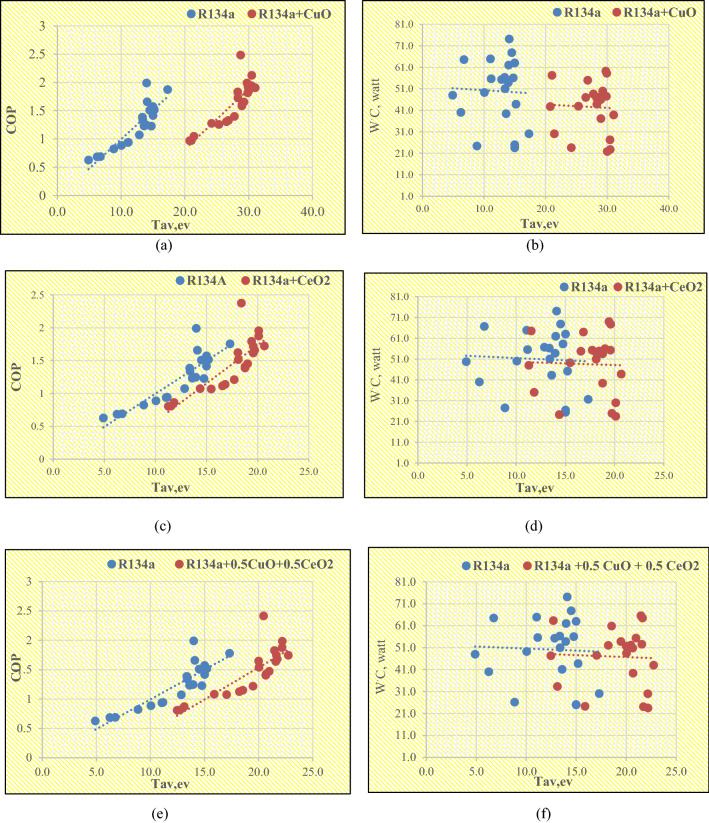

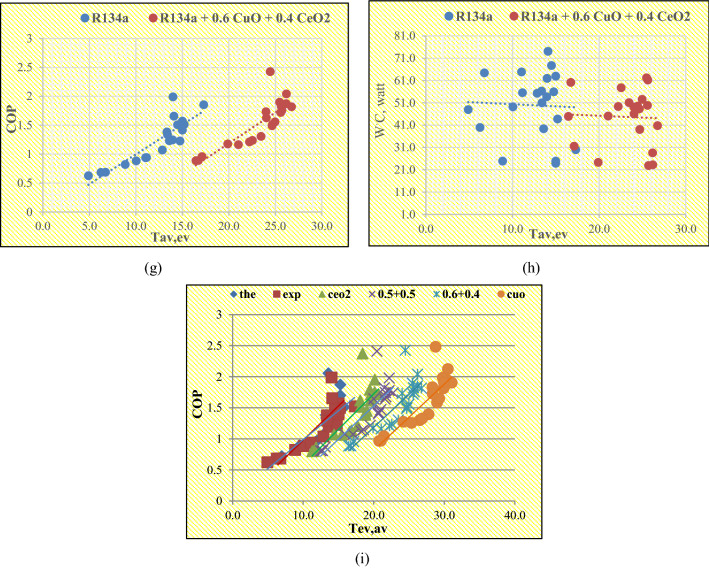
The effects of Tav, ev on Cop and WC at a constant mass fraction and comparison of the results of VCRS with nano and without nanoparticles.

The results obtained from adding CuO are illustrated graphically in Fig. 8a–i to show the effects of the average temperature of the evaporator on the COP, and WC. As it appears from the results the addition of 0.25 wt% CuO caused an increase in the temperature of the evaporator at its entrance and exit, which led to a rise in both the COP and the amount of heat absorbed inside the evaporator and thus a decrease in the amount of energy consumed by the compressor This conclusion is consistent with previous studies, and the main reason for the occurrence of these changes is the high thermal conductivity of the refrigerant due to its mixture with CuO, which records an average thermal conductivity from 20 to 40 (W/mK).The addition of the same amount of CeO_2_ caused an increase in the temperature of the evaporator at its entrance and exit, which led to a rise in both the COP and the amount of heat absorbed inside the evaporator and thus a decrease in the amount of energy consumed inside the compressor. The main reason for the occurrence of these changes is the high thermal conductivity of the refrigerant due to its mixture with CeO_2_, which records an average thermal conductivity of 11.7 (W/mK). The addition of the same amount of 0.5% CuO with 0.5% CeO_2_ caused an increase in the temperature of the evaporator at its entrance and exit, which led to a rise in both the COP and the amount of heat absorbed inside the evaporator. Thus a decrease in the amount of energy consumed inside the compressor and the main reason for the occurrence of these changes is the high thermal conductivity of the refrigerant due to its mixture with nanoparticles. The addition of the same amount of 0.6% CuO with 0.4% CeO_2_ caused an increase in the temperature of the evaporator at its entrance and exit, which led to a rise in both the COP and the amount of heat absorbed inside the evaporator and thus a decrease in the amount of energy consumed inside the compressor. The main reason for the occurrence of these changes is the high thermal conductivity of the refrigerant due to its mixture with nanoparticles. Since the most important factor in improving the performance of the refrigeration system after adding the nanoparticles is the temperature of the evaporator, all the results were plotted so that the effect of the average evaporator temperature on the COP of the system at a specific amount of nanoparticles is shown. Nagaraju et al.^31^, proved that adding copper oxide to R134a improved the COP of the refrigeration system to a degree close to what was found in this study, and the method in which nanoparticles are prepared, the shape, diameter, and quantity added to the refrigerants play an important role in determining the result.

### The results obtained from suitable models to determine the thermophysical properties of R134a

**Figure 9:**
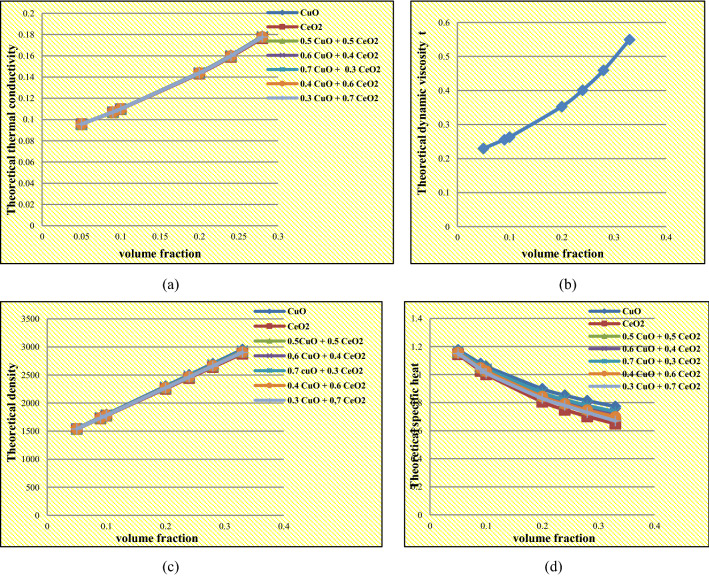
The effects of nanoparticles on the thermophysical properties of the nanorefrigerant.

The results obtained from suitable models from existing studies to determine the thermal conductivity, viscosity, density and specific heat of the nanorefrigerants for the nanoparticle concentrations of 0.05 to 0.33 vol% suspended in R-134a, are illustrated graphically in Fig. 9 and indicated that, the thermal conductivity, viscosity, and density of all types of nanoparticles increased linearly with nanoparticle volume concentration, where CuO/R-134a nanorefrigerant recorded the best value of thermal conductivity. While the specific heat decreased linearly with increasing nanoparticle volume concentration, this result is consistent with the result obtained from Alawi and Sidik^45^, who confirmed that increasing the concentration of CuO into R134a improves the thermal conductivity, viscosity and density, while the specific heat decreases.

## Conclusions

A new concept of nanoparticles was introduced to open the door for answering many questions in the future, because cerium oxide was used with copper oxide as one material consisting of a mixture of both oxides, and the results obtained for copper oxide agreed with previous studies, where copper oxide succeeded in improving the performance of the refrigeration system and increased COP by 25%, and cerium oxide succeeded in improving the performance of the system by a lesser value. For the mixture, the results confirmed that the mixture containing a higher percentage of copper oxide gave better results. The method of preparing nanoparticles was simple and affordable and produced two types of oxides and five types of mixtures. Subsequently, the field of research remains open to whether this method will succeed in obtaining other oxides, especially oxides with high thermal conductivity, because the cost of nanoparticles increases as their thermal conductivity increases. Nevertheless, the theoretical results in this research encourage researchers to move forward in the field of experimental studies This study recommends conducting experiments to verify the behavior of cerium oxide in refrigeration systems and to monitor its behavior at different temperatures for the evaporator, especially because the results of this research show that cerium oxide improved the performance of the refrigeration system due to its good thermal conductivity This study recommends mixing materials that have been prepared with other refrigerants and compressor lubricant oils to study their effect on the thermophysical properties of refrigerants and oils. Since the problem of the stability of nanoparticles with refrigerants is one of the most important problems, a hybrid consisting of different oxides was recently used to solve this problem. Will the mixture that was referred to in this study and prepared from two different oxides succeed in obtaining better results, since the mixture consisting of 50% copper oxide and 50% cerium oxide has already been mixed with R134a in the lab using the ultrasonic machine only for one hour, the result was a stable mixture for a whole day.
